# A multivariate multi-step LSTM forecasting model for tuberculosis incidence with model explanation in Liaoning Province, China

**DOI:** 10.1186/s12879-022-07462-8

**Published:** 2022-05-23

**Authors:** Enbin Yang, Hao Zhang, Xinsheng Guo, Zinan Zang, Zhen Liu, Yuanning Liu

**Affiliations:** 1 College of Computer Science and Technology, Jilin University, Changchun, 130012 China; 2 Key Laboratory of Symbolic Computation and Knowledge Engineering of Ministry of Education, Jilin University, Changchun, 130012 China; 3 College of Software, Jilin University, Changchun, 130012 China; 4 Graduate School of Engineering, Nagasaki Institute of Applied Science, 536 Aba-machi, Nagasaki, 851-0193 Japan

**Keywords:** Tuberculosis, Machine learning, Multivariate multi-step LSTM model, Hybrid forecasting model, Model explanation, SHAP

## Abstract

**Background:**

Tuberculosis (TB) is the respiratory infectious disease with the highest incidence in China. We aim to design a series of forecasting models and find the factors that affect the incidence of TB, thereby improving the accuracy of the incidence prediction.

**Results:**

In this paper, we developed a new interpretable prediction system based on the multivariate multi-step Long Short-Term Memory (LSTM) model and SHapley Additive exPlanation (SHAP) method. Four accuracy measures are introduced into the system: Root Mean Square Error, Mean Absolute Error, Mean Absolute Percentage Error, and symmetric Mean Absolute Percentage Error. The Autoregressive Integrated Moving Average (ARIMA) model and seasonal ARIMA model are established. The multi-step ARIMA–LSTM model is proposed for the first time to examine the performance of each model in the short, medium, and long term, respectively. Compared with the ARIMA model, each error of the multivariate 2-step LSTM model is reduced by 12.92%, 15.94%, 15.97%, and 14.81% in the short term. The 3-step ARIMA–LSTM model achieved excellent performance, with each error decreased to 15.19%, 33.14%, 36.79%, and 29.76% in the medium and long term. We provide the local and global explanation of the multivariate single-step LSTM model in the field of incidence prediction, pioneering.

**Conclusions:**

The multivariate 2-step LSTM model is suitable for short-term prediction and obtained a similar performance as previous studies. The 3-step ARIMA–LSTM model is appropriate for medium-to-long-term prediction and outperforms these models. The SHAP results indicate that the five most crucial features are maximum temperature, average relative humidity, local financial budget, monthly sunshine percentage, and sunshine hours.

**Supplementary Information:**

The online version contains supplementary material available at 10.1186/s12879-022-07462-8.

## Background

Tuberculosis (TB) is a respiratory infectious disease caused by Mycobacterium tuberculosis in the lungs. Its existence seriously endangers the safety of people around the world. The World Health Organization (WHO) reports the *Global Tuberculosis Report* in 2019 shows that the global situation of TB is still severe. In 2018, the number of TB cases worldwide reached 70 million, and the number of deaths from TB reached 1.5 million [1]. This number has exceeded the number of HIV/AIDS deaths. The Chinese Center for Disease Control and Prevention (CDC) classifies TB as a Class B infectious disease (the most serious is Class A). CDC statistics indicate that China has effectively controlled the spread of TB in the past decade. From 2010 to 2020, TB cases declined from 991,350 to 599,587, and the number of deaths from 3000 to 965 between January to August [2]. However, the incidence of TB in 2019 was 55.55 (1/100,000), far more than other respiratory infectious diseases. Traditional TB detection commonly uses interferon-gamma release assay (IGRA), Mantoux, and X-rays for suspected cases, which faces enormous costs and low efficiency [3, 4]. It can also be affected by a series of potential factors to contract TB, such as the sanitary conditions and ventilation of the area of residence. By providing accurate monthly results for the prevention and control center, it is possible to estimate the scale of the pandemic and thereby allocate medical resources within a region [5, 6].

The traditional forecasting models are mostly based on one-dimensional time series, including the Autoregressive Integrated Moving Average (ARIMA), gray model, and Markov model, etc. The ARIMA model, as a basic model, is widely used to predict the incidence of various infectious diseases, such as influenza [7], bacillary dysentery [8], tuberculosis [9], AIDS [10], and has been proven it is feasible and effective. When TB has seasonal characteristics, using Seasonal ARIMA (SARIMA) model can achieve better results [11, 12]. ARIMAX is a better option when the disease is influenced by a range of potential factors [13]. X is a multivariate input. In recent years, more and more studies attempted to apply Machine Learning (ML) models to forecast incidence, such as Nonlinear Autoregressive (NAR) neural network, General Regression Neural Network (GRNN), Support Vector Machine (SVM) [14]. Many studies have proved that the accuracy of ML models is generally higher than traditional models. To further improve the performance, the hybrid forecasting model has become a research hotspot [15, 16]. Researchers have been keen to discover the relationship between diseases and factors, such as meteorological factors economic and social factors [17], age and professional structure. Feature selection methods are applied to select the most crucial factors, including variance selection [18], Random Forest [19], correlation coefficient [20], Least Absolute Shrinkage and Selection Operator (LASSO) [21], etc.

Traditional forecasting models have poor adaptability to big fluctuation data. Long Short-Term Memory (LSTM) has proven to be a reliable model for forecasting the incidence of diseases [22, 23]. The unique forget gate structure may make the model perform well in the long-term prediction. Using a multivariate multistep LSTM model may obtain better prediction results. “multivariate” can mine the incidence features, and “multi-step” can alleviate seasonal and increasing trends [24]. The interpretability of ML models is a research hotspot, and its mainstream methods include Local Interpretable Model-agnostic Explanations (LIME) and SHapley Additive exPlanation (SHAP) [25]. SHAP has been used to explain the prediction of Intensive Care Unit (ICU) patient death and nitrogen dioxide concentration [26, 27]. We used the SHAP method since it can provide both local and global interpretation. Interestingly, in many ML models for incidence, no research explained why a particular model achieves excellent performance.

This paper aims to develop a model which is applicable and interpretable for predicting the incidence of TB. The LASSO and Random Forest were used to filter the features and obtain a dataset of a specified size. Then, the ARIMA, SARIMA, multivariate multi-step LSTM, and multi-step ARIMA–LSTM hybrid models were established. Through the Root Mean Square Error (RMSE), Mean Absolute Error (MAE), Mean Absolute Percentage Error (MAPE), and symmetric Mean Absolute Percentage Error (sMAPE), the performance of each model is evaluated from short (6-steps ahead), medium (12-steps ahead), and long (24-steps ahead) terms. Finally, we used the SHAP to explain the prediction results of the multivariable single-step LSTM model.

## Results

**Fig. 1:**
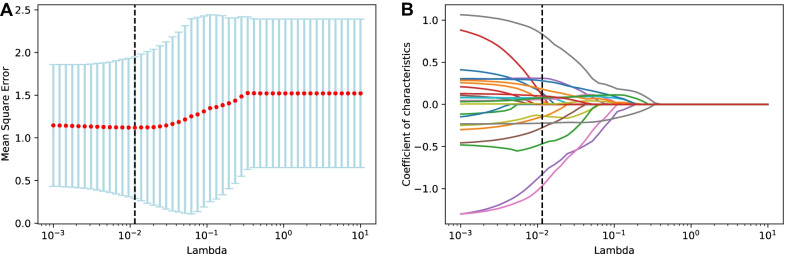
**a** Finding optimal value of $\lambda$ in LASSO by MSE. **b** Compressing the coefficient of irrelevant factor to 0 by LASSO

Through Eqs. 1 and 2, Mean Square Error (MSE) is used for cross-validation, as shown in Fig. 1a. The results show that the optimal $\lambda$ value in LASSO is 0.0202. LASSO eliminated 7 irrelevant factors, thereby reducing the factor set size from 24 to 17. The coefficient compression process is displayed in Fig. 1b. On this basis, we hope to obtain more small-sized sets of factors and analyze the changes they bring to the model performance. The procedure of Random Forest was run 5 times, the average value was taken as the importance score of each feature, and the top 10 and top 5 features were selected as the new factor sets. We thus get the factor sets with sizes 24, 17, 10, and 5.

**Fig. 2:**
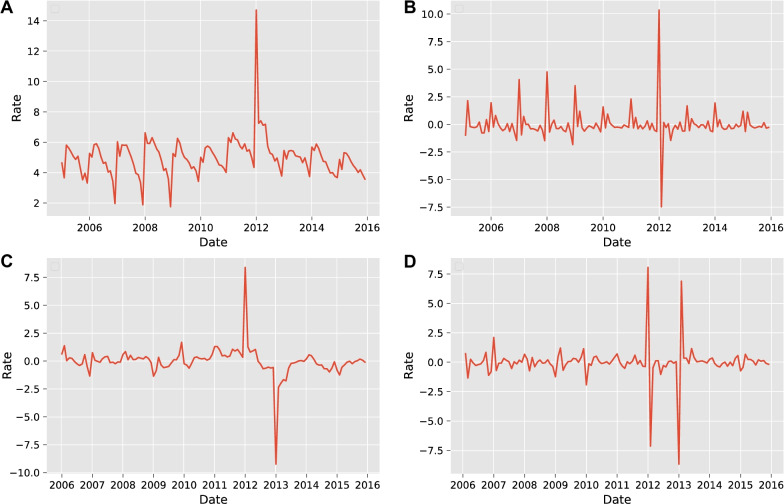
**a** Monthly incidence of TB in Liaoning Province from Jan 2005 to Dec 2015. **b** First-order differential. **c** First-order seasonal difference. **d** First-order difference and first-order seasonal difference

Figure 2a indicates that the period from January to July is the high incidence area of TB, while from October to December is the low incidence area. In general, the incidence of TB in Liaoning Province is a seasonal and periodic bimodal distribution.

The Augmented Dickey–Fuller (ADF) test shows that the p-value of the original time series is 0.50, which is greater than 0.05. Hence the series is not smooth. However, the series after first-order and first-order seasonal differencing reached smoothness with p-values of $1.94\times 10^{-6}$ and 0.01, respectively, both less than 0.05, as shown in Fig. 2b and c. Figure 2d indicates that the series becomes smoother with a p-value of $7.31\times 10^{-4}$ after first differencing and then first-order seasonal differencing. We set the parameter *d* of the ARIMA model to 1 since the series is just reaching smoothness at this point. It is unnecessary to do more differencing. Then, *p* and *q* are given as integers from 0 to 5, and a grid search is run for the model parameters (*p*, 1, *q*). Ten models with the lowest Akaike Information Criterion (AIC) and Bayesian Information Criterion (BIC) are calculated separately, and then the five optimal models are extracted from their intersection sets. The performance of these models is shown in Table 1. Similarly, five alternative SARIMA models are given in Table 2.

**Fig. 3:**
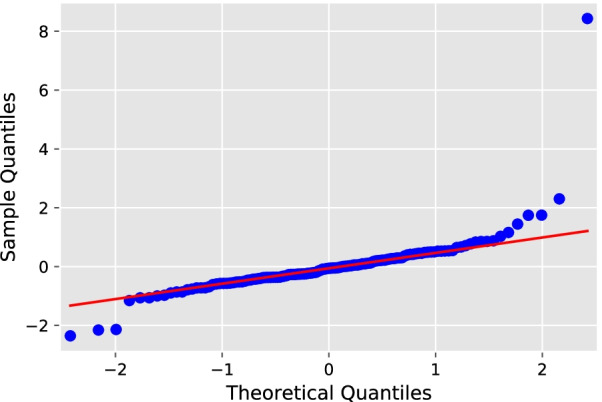
QQ plot of the ARIMA (2, 1, 4) model.

**Fig. 4:**
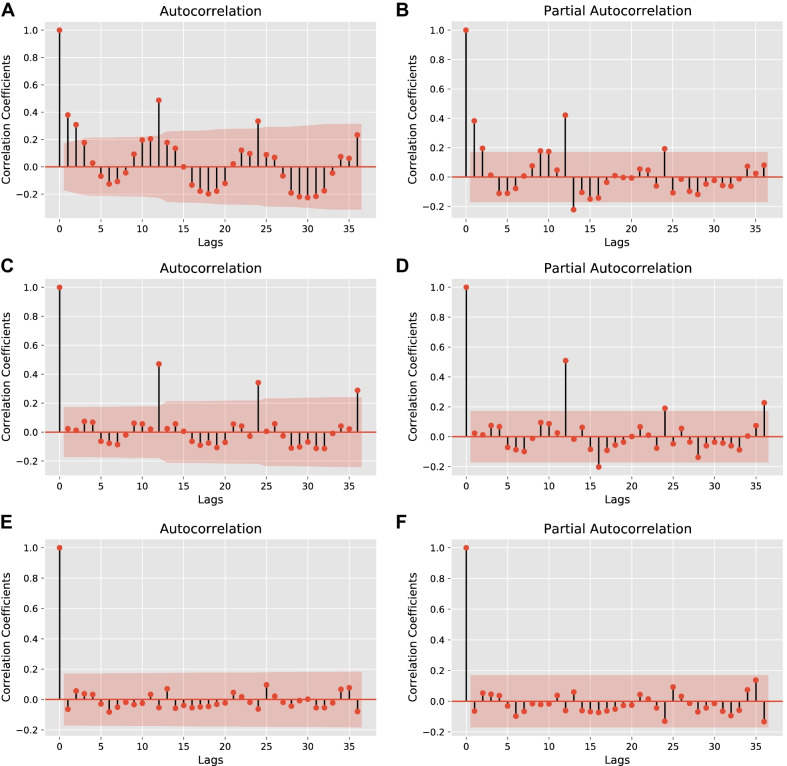
**a**, **c**, and **e** are autocorrelation plots of the original series, ARIMA (2, 1, 4) model, and SARIMA $(1, 1, 1) \times (0,1,1)_{12}$ model. **b**, **d**, and **f** are the corresponding partial autocorrelation

**Fig. 5:**
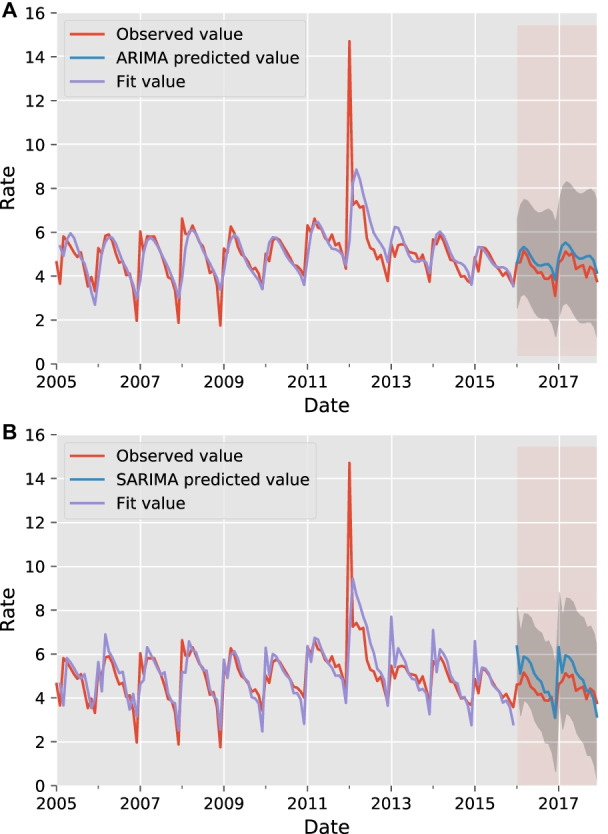
**a** ARIMA (2, 1, 4) model prediction. **b** SARIMA $(1, 1, 1) \times (0,1,1)_{12}$ model prediction

The test results of the ARIMA (2,1,4) model indicated that the distribution of points in the Quantile–Quantile (QQ) plot (as in Fig. 3) was along 45°, as well as the Durbin–Watson (D–W) value was 1.94, which is close to 2, and the p-value was 0.79, which is greater than 0.05, proving that the model applies to this study. From Fig. 4a and b, the TB incidence has a clear cyclical pattern. The autocorrelation coefficients of the optimal ARIMA model are not all fall within the confidence interval (Fig. 4c and d), while the optimal SARIMA model does. Theoretically, the SARIMA model should be more suitable for prediction (Fig. 4e and f). However, the MAPE results indicate that ARIMA (2, 1, 4) is the best model. It is considered that a MAPE value less than 10% is an accurate forecasting model [28]. The MAPE value of the best SARIMA model is 14.1518, which has a poor performance. The prediction results of the two models are shown in Fig. 5a and b.

**Table 1:** A series of alternative the ARIMA (*p*, 1, *q*) models (24 steps ahead prediction)

*(p, q)*	AIC	BIC	RMSE	MAE	MAPE (%)	sMAPE (%)
(5, 5)	408.04	442.55	0.5628	0.4724	11.1506	10.3224
(3, 3)	419.26	442.26	1.3423	1.1488	26.5904	22.5749
$\varvec{(2, 4)}$	**419.61**	**442.62**	**0.4672**	**0.4177**	**9.9328**	**9.3198**
(2, 1)	427.84	442.21	0.8994	0.7413	16.9383	19.3824
(3, 5)	417.13	445.88	0.9290	0.7617	17.8239	15.7419

Bold indicates the best performing model

**Table 2:** A series of alternative the SARIMA $(p, 1, q) \times {(P, 1, Q)_{12}}$ models (24 steps ahead prediction)

*(p, q, P, Q)*	AIC	BIC	RMSE	MAE	MAPE (%)	sMAPE (%)
(0, 1, 0, 1)	340.64	348.98	0.8471	0.7204	15.9791	14.5258
$\varvec{(1, 1, 0, 1)}$	**341.49**	**352.60**	**0.7634**	**0.6384**	**14.1518**	**13.0495**
(0, 2, 0, 1)	341.56	352.68	0.7778	0.6519	14.4502	13.2851
(0, 1, 1, 1)	342.54	353.66	0.8323	0.7083	15.7193	14.3164
(1, 2, 0, 1)	342.83	356.73	0.8040	0.6764	14.9867	13.7548

Bold indicates the best performing model

**Table 3:** Forecast performance of the multivariate LSTM model with different size factor sets

Number of factors	RMSE	MAE	MAPE (%)	sMAPE (%)
24	0.5002	0.3779	9.1007	8.7484
**17**	**0.4499**	**0.3398**	**8.3577**	**7.9290**
10	0.4854	0.3502	8.6350	8.0442
5	0.6184	0.4462	11.1905	9.9987
0	0.5213	0.4106	10.2265	9.4457

Bold indicates the best performing model

**Fig. 6:**
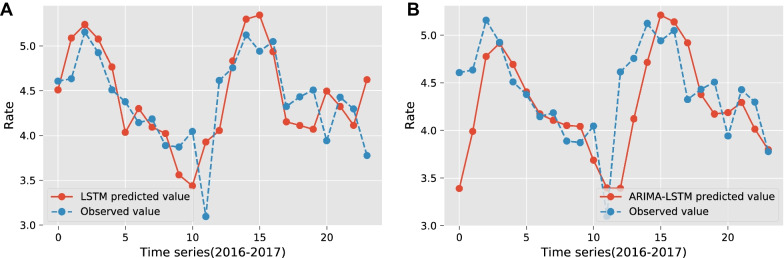
**a** Multivariate 2-step LSTM model prediction. **b** 3-step ARIMA–LSTM hybrid model prediction

The LSTM solver was run 10 times and the average value was taken as the final error result. Table 3 indicates that the data set, filtered by LASSO only, of size 17, is the optimal factor set. The improvement for model performance is better than the sets of sizes 24, 10, and 5, without adding the step. It also outperforms the traditional LSTM model based on time series only (0 factors). Finally, we solved the multivariate multi-step LSTM models with step sizes of 1, 2, 3, and 4, respectively, and observed the best prediction results at 2-step. Compared with the ARIMA model, the multivariate 2-step LSTM model has better performance at any stage, as in Fig. 6a, with a reduction in each error ranging from 8.25 to 22.04%, as presented in Table 4.

Similarly, we selected the same range of step sizes as the LSTM model, thus determining the optimal step size for the hybrid model was 3-step. The 3-step ARIMA–LSTM hybrid model performs poorer than the ARIMA model in the short-term forecast. However, the hybrid model demonstrated excellent performance in the medium and long-term forecasts, as in Fig. 6b. The reduction in each error compared to the optimal ARIMA model ranges from 13.48 to 36.79%. It means the performance is nearly double of the multivariate 2-step LSTM model.

**Fig. 7:**
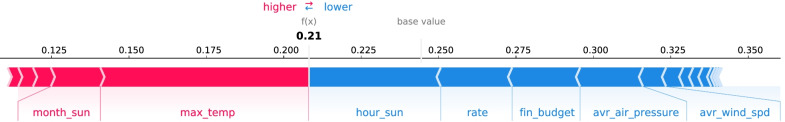
Impact of single sample characteristics (January 2016 forecast)

**Fig. 8:**
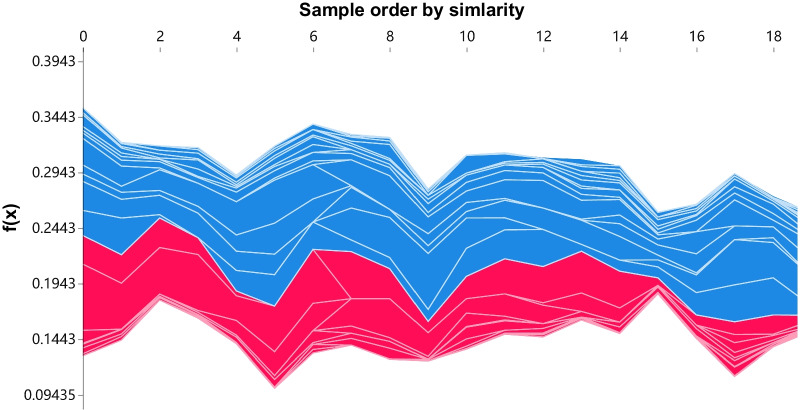
Feature impact (24 samples of the test set)

We make the first attempt to explain ML forecasting models in the field of infectious disease incidence prediction. The multivariate single-step LSTM model is taken as an example, and the SHAP method is applied for model interpretation. A single sample from January 2016 was selected for the local explanation, and the results are shown in Fig. 7. Features in red indicate pushing the predicted value higher, and features in blue mean driving it lower. Each feature pushes the predicted value of the model, from $base \ value=0.25$ to $f(x)=0.21$. All 24 samples of the test set can be expressed, giving a feature impact map, as displayed in Fig. 8. Each line represents a feature, the horizontal axis indicates the number of samples, and the vertical axis denotes the SHAP value of the feature.

**Fig. 9:**
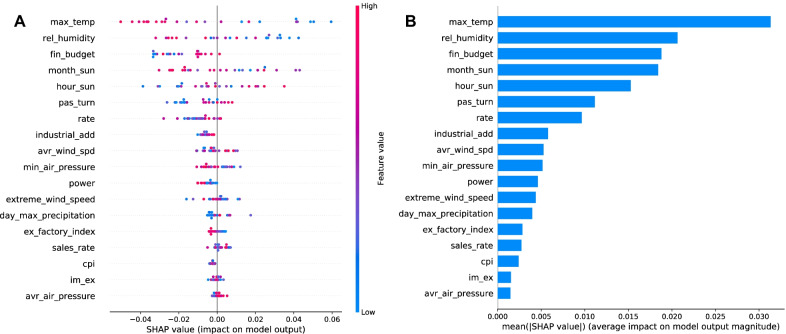
**a** Scatter plot of feature density. **b** Feature importance SHAP values

The 24 samples of the test set, from January 2016 to December 2017, were selected for the global explanation, and the feature density scatter plot was drawn as shown in Fig. 9a. The horizontal axis is the SHAP value, and each row represents one feature. Red and blue indicate high and low feature values, respectively. To be specific, the first feature, *max_temp*, means the maximum temperature, and the results show that its lower value will instead drive up the predicted value of TB incidence. The fifth feature, *hour_sun*, indicates the monthly sunshine hours, and its higher value pushes up the predicted value. Finally, the scatter of the last five features oscillates around the SHAP value equal to 0, and there is no spread to either side, which suggests that these features are not associated with the predicted values. The absolute value of SHAP value was first taken and then averaged to serve as the feature importance, as illustrated in Fig. 9b. The five most important features affecting the incidence of TB in Liaoning Province are maximum temperature (*max_temp*), average relative humidity (*rel_humidity*), local financial budget (*fin_budget*), monthly sunshine percentage (*month_sun*), and monthly sunshine hours (*hour_sun*).

## Discussion

This study aimed to design accurate forecasting models for TB incidence to serve as a reference for epidemic prevention and control departments in Liaoning province. At any stage, using a multivariate 2-step LSTM model can reduce the error by 8.25% to 22.04%, and this performance is on the same level as similar studies. In medium and long-term prediction, if pursuing higher accuracy, the 3-step ARIMA–LSTM hybrid model can be used, and each error can be reduced by 13.48% to 36.79%, an effect that is higher than similar studies over 10% to 20%.

The ARIMA model, as a baseline, is valid for the majority of infectious disease predictions and is therefore widely used as a comparison to evaluate the performance of new models. The SARIMA model was proved to be an unreasonable model in predicting the monthly incidence of TB, and in the short term, the error value was even three times higher than the optimal ARIMA model, probably due to the less significant seasonal incidence characteristics of the region.

**Table 4:** Comparison of the forecast performance of each model

Model	RMSE	MAE	MAPE (%)	sMAPE (%)
6-step ahead prediction between January 2016 to June 2016				
*A*	0.3244 (−)	0.2811 (−)	6.0454 (−)	5.8097 (−)
*B*	1.0157 (+ 213.10%)	0.9339 (+ 232.23%)	20.0035 (+ 230.89%)	17.9006 (+ 208.12%)
$\varvec{C}^{\star }$	**0.2825 (− 12.92%)**	**0.2363 (− 15.94%)**	**5.0797 (− 15.97%)**	**4.9490 (− 14.81%)**
$D^{\star }$	0.4659 (+ 43.62%)	0.3206 (+ 14.05%)	6.8661 (+ 13.58%)	7.4156 (+ 27.64%)
12-step ahead prediction between January 2016 and December 2016				
*A*	0.4425 (−)	0.3917 (−)	9.7674 (−)	9.1462 (−)
*B*	0.7825 (+ 63.40%)	0.6508 (+ 66.15%)	14.5400 (+ 48.86%)	13.2301 (+ 44.65%)
$C^{\star }$	0.4060 (− 8.25%)	0.3073 (− 21.55%)	7.8076 (− 20.06%)	7.5203 (− 17.78%)
$\varvec{D}^{\star }$	**0.3753 (− 15.19%)**	**0.2619 (− 33.14%)**	**6.1742 (− 36.79%)**	**6.4240 (− 29.76%)**
24-step ahead prediction between January 2016 and December 2017				
*A*	0.4672 (−)	0.4177 (−)	9.9328 (−)	9.3198 (−)
*B*	0.7634 (+ 63.40%)	0.6384 (+ 52.84%)	14.1518 (+ 42.48%)	13.0495 (+ 40.02%)
$C^{\star }$	0.4108 (− 12.07%)	0.3295 (− 21.12%)	7.7436 (− 22.04%)	7.4895 (− 19.64%)
$\varvec{D}^{\star }$	**0.4042 (− 13.48%)**	**0.3070 (− 26.50%)**	**6.9664 (− 29.86%)**	**7.2245 (− 22.48%)**

The data format *x*(*y*), *x* is the error value and *y* is the percentage change compared to the ARIMA model. Particularly, (–) indicates the null value. *A* is the ARIMA model and *B* is the SARIMA model. The new model proposed in this paper is labeled by superscript $\star$. $C^{\star }$ is the multivariate 2-step LSTM model and $D^{\star }$ is the 3-step ARIMA–LSTM hybrid forecasting model

There is no fixed incidence trend of monthly incidence of TB in Liaoning Province. For instance, the incidence rate decreased and then increased from January to March from 2013 to 2015, while it showed a linear downward trend from April to July. The trends did not follow the previous pattern from January to July between 2016 and 2017. However, the applicability of the SARIMA model for TB cannot be denied, and some such studies have still given favorable conclusions.

The factor set size of 17 had the most significant improvement in the prediction of the LSTM model and can be considered to contain the most important features influencing TB. The set size of 24 contained excessive irrelevant factors. In other words, there was noise in the data, which led to poor performance. The factor sets of sizes 10 and 5 lack important features, and the traditional LSTM model is based only on time series, so they do not improve prediction accuracy.

The suitable input step size mitigates the increasing trend of incidence. Thus, the forecast performance of the multivariate LSTM model is further improved. In long-term forecasting, the multivariate 2-step LSTM model still achieves better effects than the ARIMA model, perhaps attributed to the unique “forget gate” structure of the LSTM, resulting in excellent memory capability for previous sequences [29, 30].

Based on the results of the SHAP explanation, we can know which are the important features and what role, positive or negative, these features play in the generation of predicted values by the model. Meteorological factors are crucial in the incidence of TB, and many studies have shown clear conclusions. That also corresponds to the SHAP results in this paper. Temperature, sunshine hours, and humidity are all positive factors, and they may increase the risk of incidence. Higher temperatures may be associated with longer sunshine hours, and under such conditions, pathogens are more likely to replicate [31]. There is an experiment demonstrating that a room with humidity higher than 75% is double the likelihood of being positive for Mycobacterium TB [32]. However, wind speed is a negative factor. Areas with lower wind speed tend to have poor ventilation and the people there are more easily infected [33, 34].

Some results cannot be proven from previous studies, but the reasons are complex and vary from region to region. SHAP results indicate that lower local financial budgets are associated with lower incidence rates. Poor and remote areas generally face a higher risk of incidence [35]. In a region, there is a strong connection between the economic situation and the population density, medical conditions, and living environment of the residents. Therefore, we are unable to determine which factor plays a dominant role. There are countless factors influencing the incidence of TB, such as age, individual’s nutritional condition, whether they smoke or have been vaccinated with anti-tuberculosis drugs, race, air pollution, and living conditions [36–38]. In conclusion, collecting more valid factors is promising for improving prediction performance.

Our study has some limitations and drawbacks. First, we selected a few factors, which were largely limited by the dataset resources. Considering more factors and regions may lead to more meaningful findings. Second, the LSTM designed in this paper has only three layers, and a more complex network structure, combined with parameter tuning methods, may obtain better performance.

The causes of TB pathogenesis are complex, so we need to consider temporal and spatial conditions sufficiently, and we can add the Graph Neural Networks (GNN) to our prediction model to realize the connection between multiple regions [39]. It may be more meaningful to assess the risk of infection in specific groups.

## Conclusions

In conclusion, the prediction accuracy of the multivariate 2-step LSTM model and the 3-step ARIMA–LSTM hybrid model are both better than that of the traditional ARIMA model and LSTM model. In particular, the hybrid models show excellent performance in the medium and long term. Furthermore, the explanation results of the ML forecasting models can lead us to pick more important features that affect the incidence of TB.

## Methods

### Data sources

Liaoning Province is located in the southern part of northeast China, between 3843′ N and 4326′ N, and between 11853′ E and 12546′ E, with a total area of 148,600 km^2^. From the Public Health Science Data Center (https://www.phsciencedata.cn), TB incidence data in Liaoning Province from 2005 to 2017, 156 months, were collected. From the National Meteorological Science Data Center (https://data.cma.cn), 15 sets of corresponding meteorological data were obtained, including temperature, humidity, sunshine duration, precipitation, pressure, etc. From the National Bureau of Statistics (https://data.stats.gov.cn), 9 economic and social data were obtained, including Consumer Price Index (CPI), number of tourists, industrial output, etc (Additional files 1, 2, 3, 4).

### Data cleaning

The data used in this paper has a good consistency and no outliers. However, some sites were missing data for several months for 24 sets of factor data. The missing values are alternately filled by the k-Nearest Neighbor (kNN) algorithm and the multivariate feature imputation. The principle of kNN is to find the “nearest” numbers of *k* samples in the data set to target and interpolate with the average value of *k* samples [40]. Such “nearest” is measured by Euclidean distance. The multivariate feature imputation is an alternative method when kNN fills in all missing values with the same value [41].

### Feature selection

Factor data were not all significantly associated with TB incidence, so it was necessary to use LASSO to remove the irrelevant factors first. To obtain a range of smaller sets of factors, we then used Random Forest to rank the feature importance. The core of LASSO is to compress the coefficient before the irrelevant variable to zero in the regression problem [42]. The regression problem in this paper can be expressed as

1 $$\begin{aligned}&{y_i} = {\omega ^T}{x_i} + b \end{aligned}$$

2 $$\begin{aligned}&J(w)= \frac{1}{m}\sum \limits _{i = 1}^m {{{({y_i} - {\omega ^T}{x_i})}^2}} + \lambda \sum \limits _{i = 1}^m {\left| {{w_i}} \right| } , \lambda > 0 \end{aligned}$$

where, $y_i$, $x_i$ and $\omega ^T$ represent the monthly incidence of TB, factors, and regression coefficients. The cost function *J*(*w*) is introduced to evaluate the accuracy of the regression model. It is necessary to find the $\lambda$ that minimizes the value of *J*(*w*).

### Division of train set and test set

For all models, 132 months of data between January 2005 and December 2015 were used as the train set. The test set selected data from January 2016 to December 2017, in which 6, 12, and 24 months after January 2016 were employed as the verification of 6, 12, and 24 steps ahead prediction.

### Building ARIMA model and SARIMA model

In the ARIMA model, “AR” represents autoregression, describing the past and present data relationship. “I” denotes differential operational. “MA” stands for moving average, which is the sum of the error terms in “AR” [43]. The ARIMA model is denoted as *ARIMA*(*p*, *d*, *q*), where *p*, *d* and *q* represent the order of autoregression, difference, and moving average. The SARIMA model is denoted as $SARIMA(p,d,q) \times {(P,D,Q)_s}$. *P*, *D*, and *Q* represent the order of seasonal model parameters, and *s* is the cycle length.

Before building, we can obtain the characteristics of the data (e.g. seasonality and periodicity) by using the autocorrelation function (ACF) and the partial autocorrelation function (PACF). On this basis, approximate p and q values are determined. ADF test was used to verify the stationarity of the time series. We used information criteria, AIC and BIC to determine the order of parameters. AIC is an index to evaluate the fitting effect of the model based on entropy, as shown in Eq. 3. BIC strengthened the punishment item and included the sample number *n* into the assessment, as shown in Eq. 4. We verify the applicability of these models according to the QQ plot, D–W test, and white noise test. After model establishment, the ACF and PACF plots of the residuals are used to evaluate whether this model is suitable for prediction.

3 $$\begin{aligned} AIC&= - 2\ln (L) + 2k \end{aligned}$$

4 $$\begin{aligned} BIC&= - 2\ln (L) + k\ln (n) \end{aligned}$$

where *L* is the likelihood function and *k* is the number of model parameters.

### A new interpretable prediction system: multivariate multi-step LSTM model and SHAP

In this section, we applied the multivariate multi-step LSTM model to predict incidence in the field of infectious diseases for the first time. The model is selected according to four error indicators. Combined with SHAP, a model explanation method, an interpretable prediction system is proposed.

**Fig. 10:**
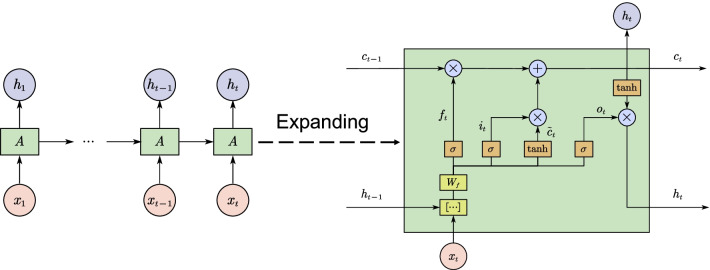
The three-layer LSTM internal and external structure used in this paper

**Fig. 11:**
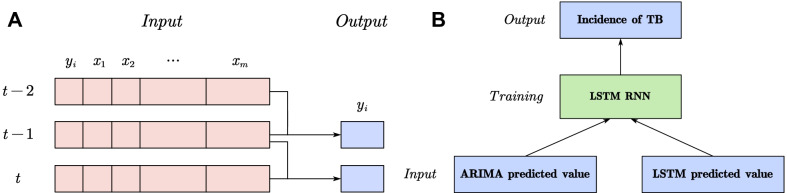
**a** Input and output of multivariate n-step LSTM model (when $n=2$). **b** Hybrid forecasting model principle

#### LSTM structure and theory

LSTM is a variant of Recurrent Neural Network (RNN). Compared with RNN, the memory ability of LSTM was significantly improved. LSTM introduces the concept of “gate”, namely forget gate, input gate, and output gate. Forget gate determines how much information about the previous cell is retained. The input of the forget gate at time *t* includes output ${h_{t - 1}}$ of the hidden layer at time $t-1$ and new input ${x_t}$, which is processed by weighting and Sigmoid activation function, as shown in Eq. 5. Input gate controls the input and update of cell state. ${f_t}$ is the extent to which previous information ${c_{t - 1}}$ is forgotten, and ${i_t}$ is also the extent to which new information ${{\tilde{c}}_t}$ is added to the cell, as shown in Eqs. 6 to 8. Then, output gate selectively outputs which parts, as shown in Eqs. 9 and 10. The three-layer LSTM structure used in this paper is illustrated in Fig. 10.

5 $$\begin{aligned}&{f_t} = sigmoid({W_f}[{h_{t - 1}},{x_t}] + {b_f}) \end{aligned}$$

6 $$\begin{aligned}&{i_t} = sigmoid({W_i}[{h_{t - 1}},{x_t}] + {b_i}) \end{aligned}$$

7 $${{{\tilde{c}}}_t} = \tanh ({W_c}[{h_{t - 1}},{x_t}])$$

8 $$\begin{aligned}&{c_t} = {f_t}{c_{t - 1}} + {i_t}{{{\tilde{c}}}_t} \end{aligned}$$

9 $$\begin{aligned}&{o_t} = sigmoid({W_o}[{h_{t - 1}},{x_t}] + {b_o}) \end{aligned}$$

10 $${h_t} = {o_t}\tanh ({c_t})$$

#### Definition of “multivariate” and “multi-step”

In the multivariate multi-step LSTM model, if numbers of *m* factors are selected, “multivariate” means that the input ${x_t}$ at time *t* is a $1 \times m$ row vector concatenated from *m* factor data. “multi-step” means that predicting the incidence at time *t* requires $t,t-1,\ldots ,t-n+1$ data as input, which is similar to the “sliding window”. *n* is the step size. The 2-step forecasting diagram is depicted in Fig. 11a. The multivariate multi-step LSTM model can fully consider the factors affecting the incidence of TB, and alleviate the increasing trend by setting multi-step, thereby may obtain a higher prediction accuracy.

#### Model selection

We introduced four error measures to assess the accuracy of these models. The RMSE and MAE were chosen as scale-dependent measures, and the MAPE and sMAPE were selected as scale-independent measures, which are calculated by Eqs. 11 to 14.

11 $$RMSE = \sqrt{\frac{1}{n}\sum \limits _{i = 1}^n {({{\hat{y}}}_i} - {y_i})^2}$$

12 $$\begin{aligned}&MAE = \frac{1}{n}\sum \limits _{i = 1}^n {\left| {{{{\hat{y}}}_i} - {y_i}} \right| } \end{aligned}$$

13 $$\begin{aligned}&MAPE = \frac{{100\% }}{n}\sum \limits _{i = 1}^n {\left| {\frac{{{{{\hat{y}}}_i} - {y_i}}}{{{y_i}}}} \right| } \end{aligned}$$

14 $$\begin{aligned}&sMAPE = \frac{{100\% }}{n}\sum \limits _{i = 1}^n {\frac{{2\left| {{{{\hat{y}}}_i} - {y_i}} \right| }}{{\left| {{{{\hat{y}}}_i}} \right| + \left| {{y_i}} \right| }}} \end{aligned}$$

where, ${{{{\hat{y}}}_i}}$ is the predicted value, ${{y_i}}$ is the observed value, and *n* is the series length.

#### Model explanation

SHapley Additive exPlanation (SHAP) is a post-hoc method of interpretation. SHAP regards all features as ‘contributors” and constructs an additive model by calculating the marginal contribution of features to the model output, as shown in Eq. 15 [44–46]. And it can explain why ML forecasting models, even some of the “black box”, make certain prediction results. Model interpretation can be divided into Local Interpretation and Global Interpretation. Local interpretation explains a single instance, or the relationship between independent and dependent variables in a data subset [47]. Global interpretation is based on the entire data set or model level [48].

15 $$\begin{aligned} {y_i} = {y_{base}} + f({x_{i1}}) + f({x_{i2}}) + \cdots + f({x_{ik}}) \end{aligned}$$

where, ${y_i}$ is the predicted value, ${y_{base}}$ is the base value of the model, and $f({x_{ik}})$ is the SHAP value of feature *k*.

### A new method: multi-step ARIMA–LSTM hybrid model

The hybrid forecasting model can combine the strengths of many models. In this paper, the ARIMA–LSTM hybrid model takes the predicted value of the optimal ARIMA model and LSTM model as input, and takes the actual value of TB incidence as the label, using the LSTM for training, as presented in Fig. 11b. We introduced multiple steps to the hybrid model, pioneering, eliminating the increasing trend of the series, thereby improving the prediction accuracy.

## Supplementary Information

**Additional file 1.** Complete data (pre-processed).

**Additional file 2.** Economic and social factors (raw data).

**Additional file 3.** Meteorological factors (raw data).

**Additional file 4.** Supplementary documentation.

## Data Availability

The data and code are available on GitHub (https://github.com/EnbinYang/tb_prediction_files).

## Abbreviations

TB: Tuberculosis
ARIMA: Autoregressive Integrated Moving Average
LSTM: Long Short-Term Memory
LASSO: Least Absolute Shrinkage and Selection Operator
kNN: k-Nearest Neighbor
SHAP: SHapley Additive exPlanation
RMSE: Root Mean Square Error
MAE: Mean Absolute Error
MAPE: Mean Absolute Percentage Error
sMAPE: Symmetric Mean Absolute Percentage Error

## References

[1] Harding E (2019). WHO global progress report on tuberculosis elimination. Lancet Respir Med.

[2] Li T, Du X, Liu X, Li Y, Zhao Y (2021). Implementation Performance of Tuberculosis Control in China: 2011–2020. China CDC Wkly.

[3] Kik SV, Franken WP, Mensen M, Cobelens FG, Kamphorst M, Arend SM, Erkens C, Gebhard A, Borgdorff MW, Verver S (2010). Predictive value for progression to tuberculosis by IGRA and TST in immigrant contacts. Eur Respir J.

[4] Rothel JS, Andersen P (2005). Diagnosis of latent Mycobacterium tuberculosis infection: is the demise of the Mantoux test imminent?. Expert Rev Anti-infect Ther.

[5] Jeffries C, Lobue P, Chorba T, Metchock B, Kashef I (2017). Role of the health department in tuberculosis prevention and control-legal and public health considerations. Microbiol Spectr.

[6] World Health Organization (2014). Infection prevention and control of epidemic-and pandemic-prone acute respiratory infections in health care.

[7] Wang C, Li Y, Feng W, Liu K, Zhang S, Hu F, Jiao S, Lao X, Ni H, Xu G (2017). Epidemiological features and forecast model analysis for the morbidity of influenza in Ningbo, China, 2006–2014. Int J Environ Res Public Health.

[8] Li G-Z, Shao F-F, Zhang H, Zou C-P, Li H-H, Jin J (2015). High mean water vapour pressure promotes the transmission of bacillary dysentery. PLoS ONE.

[9] Wang K, Deng C, Li J, Zhang Y, Li X, Wu M (2017). Hybrid methodology for tuberculosis incidence time-series forecasting based on ARIMA and a NAR neural network. Epidemiol Infect.

[10] Li Z, Li Y (2020). A comparative study on the prediction of the BP artificial neural network model and the ARIMA model in the incidence of AIDS. BMC Med Inform Decis Mak.

[11] Zhang G, Huang S, Duan Q, Shu W, Hou Y, Zhu S, Miao X, Nie S, Wei S, Guo N (2013). Application of a hybrid model for predicting the incidence of tuberculosis in Hubei, China. PLoS ONE.

[12] Ding Z, Li Y, Wang X, Li H, Wang W (2020). The impact of air pollution on the transmission of pulmonary tuberculosis. Math Biosci Eng.

[13] Li Z-Q, Pan H-Q, Liu Q, Song H, Wang J-M (2020). Comparing the performance of time series models with or without meteorological factors in predicting incident pulmonary tuberculosis in eastern China. Infect Dis Poverty.

[14] Siriyasatien P, Phumee A, Ongruk P, Jampachaisri K, Kesorn K (2016). Analysis of significant factors for dengue fever incidence prediction. BMC Bioinform.

[15] Yuan C, Liu S, Fang Z (2016). Comparison of China’s primary energy consumption forecasting by using ARIMA (the autoregressive integrated moving average) model and GM(1,1) model. Energy.

[16] Wang Y, Xu C, Li Y, Wu W, Gui L, Ren J, Yao S (2020). An advanced data-driven hybrid model of SARIMA-NNNAR for tuberculosis incidence time series forecasting in Qinghai Province, China. Infect Drug Resist.

[17] Dean HD, Fenton KA (2010). Addressing social determinants of health in the prevention and control of HIV/AIDS, viral hepatitis, sexually transmitted infections, and tuberculosis. Public Health Rep.

[18] Yang C, Zhang W, Zou J, Hu S, Qiu J (2013). Feature selection in decision systems: a mean-variance approach. Math Probl Eng.

[19] Zhou Q, Zhou H, Li T (2016). Cost-sensitive feature selection using random forest: Selecting low-cost subsets of informative features. Knowl-based Syst.

[20] Ge R, Zhou M, Luo Y, Meng Q, Mai G, Ma D, Wang G, Zhou F (2016). McTwo: a two-step feature selection algorithm based on maximal information coefficient. BMC Bioinform.

[21] Ghosh P, Azam S, Jonkman M, Karim A, Shamrat FJM, Ignatious E, Shultana S, Beeravolu AR, De Boer F (2021). Efficient prediction of cardiovascular disease using machine learning algorithms with relief and LASSO feature selection techniques. IEEE Access.

[22] Gu J, Liang L, Song H, Kong Y, Ma R, Hou Y, Zhao J, Liu J, He N, Zhang Y (2019). A method for hand-foot-mouth disease prediction using GeoDetector and LSTM model in Guangxi, China. Sci Rep.

[23] Chae S, Kwon S, Lee D (2018). Predicting infectious disease using deep learning and big data. Int J Environ Res Public Health.

[24] Brownlee J. Deep learning for time series forecasting: predict the future with MLPs, CNNs and LSTMs in Python. Machine Learning Mastery; 2018. p. 123–160.

[25] Lombardi A, Diacono D, Amoroso N, Monaco A, Tavares JMR, Bellotti R, Tangaro S (2021). Explainable deep learning for personalized age prediction with brain morphology. Front Neurosci.

[26] Thorsen-Meyer H-C, Nielsen AB, Nielsen AP, Kaas-Hansen BS, Toft P, Schierbeck J, Strøm T, Chmura PJ, Heimann M, Dybdahl L (2020). Dynamic and explainable machine learning prediction of mortality in patients in the intensive care unit: a retrospective study of high-frequency data in electronic patient records. Lancet Digit Health.

[27] García MV, Aznarte JL (2020). Shapley additive explanations for NO2 forecasting. Ecol Inform.

[28] Wang Y, Xu C, Zhang S, Wang Z, Yang L, Zhu Y, Yuan J (2019). Temporal trends analysis of tuberculosis morbidity in mainland China from 1997 to 2025 using a new SARIMA-NARNNX hybrid model. BMJ Open.

[29] Shao X, Kim CS (2020). Multi-step short-term power consumption forecasting using multi-channel LSTM with time location considering customer behavior. IEEE Access.

[30] Song X, Liu Y, Xue L, Wang J, Zhang J, Wang J, Jiang L, Cheng Z (2020). Time-series well performance prediction based on Long Short-Term Memory (LSTM) neural network model. J Pet Sci Eng.

[31] Gelaw YA, Yu W, Magalhães RJ, Assefa Y, Williams G (2019). Effect of temperature and altitude difference on tuberculosis notification: a systematic review. J Glob Infect Dis.

[32] Lestari P, Sustini F, Endaryanto A, Setyoningrum RA (2016). Home humidity increased risk of tuberculosis in children living with adult active tuberculosis cases. J Univ Med.

[33] Amsalu E, Liu M, Li Q, Wang X, Tao L, Liu X, Luo Y, Yang X, Zhang Y, Li W, Li X (2019). Spatial-temporal analysis of tuberculosis in the geriatric population of China: an analysis based on the Bayesian conditional autoregressive model. Arch Gerontol Geriatr.

[34] Gao C, Wang Y, Hu Z, Jiao H, Wang L (2022). Study on the associations between meteorological factors and the incidence of pulmonary tuberculosis in Xinjiang, China. Atmosphere.

[35] World Health Organization (2016). World malaria report 2015.

[36] Nava-Aguilera E, Andersson N, Harris E, Mitchell S, Hamel C, Shea B, López-Vidal Y, Villegas-Arrizón A, Morales-Pérez A (2009). Risk factors associated with recent transmission of tuberculosis: systematic review and meta-analysis. Int J Tuberc Lung Dis.

[37] Kurmi OP, Sadhra CS, Ayres JG, Sadhra SS (2014). Tuberculosis risk from exposure to solid fuel smoke: a systematic review and meta-analysis. J Epidemiol Community Health.

[38] Lin HH, Ezzati M, Murray M (2007). Tobacco smoke, indoor air pollution and tuberculosis: a systematic review and meta-analysis. PLoS Med.

[39] Fan J, Bai J, Li Z, Ortiz-Bobea A, Gomes CP. A GNN-RNN approach for harnessing geospatial and temporal information: application to crop yield prediction. arXiv preprint. 2021. arXiv:2111.08900.

[40] Troyanskaya O, Cantor M, Sherlock G, Brown P, Hastie T, Tibshirani R, Botstein D, Altman RB (2001). Missing value estimation methods for DNA microarrays. Bioinformatics.

[41] Van Buuren S, Groothuis-Oudshoorn K (2011). mice: Multivariate imputation by chained equations in R. J Stat Softw.

[42] Tibshirani R (1996). Regression shrinkage and selection via the lasso. J R Stat Soc Ser B.

[43] Kırbaş İ, Sözen A, Tuncer AD, Kazancıoğlu FŞ (2020). Comparative analysis and forecasting of COVID-19 cases in various European countries with ARIMA, NARNN and LSTM approaches. Chaos Solitons Fractals.

[44] Butnariu D (1980). Stability and Shapley value for an n-persons fuzzy game. Fuzzy Sets Syst.

[45] Lundberg SM, Lee S-I. A unified approach to interpreting model predictions. In: Proceedings of the 31st international conference on neural information processing systems. 2017;4768–77.

[46] Ribeiro MT, Singh S, Guestrin C. ” Why should i trust you?” Explaining the predictions of any classifier. In: Proceedings of the 22nd ACM SIGKDD international conference on knowledge discovery and data mining. 2016;1135–44.

[47] Liang Y, Li S, Yan C, Li M, Jiang C (2021). Explaining the black-box model: a survey of local interpretation methods for deep neural networks. Neurocomputing.

[48] Díaz G, Coto J, Gómez-Aleixandre J (2019). Prediction and explanation of the formation of the Spanish day-ahead electricity price through machine learning regression. Appl Energy.

