# Supplementary material for: A multivariate multi-step LSTM forecasting model for tuberculosis incidence with model explanation in Liaoning Province, China
Source: BMC Infect Dis. 2022 May 23;22:490. doi: 10.1186/s12879-022-07462-8 (PMC9128107; doi:10.1186/s12879-022-07462-8)
Supplement: Supplementary file 4 — Additional file 4. Supplementary documentation. [file 12879_2022_7462_MOESM4_ESM.pdf]

A multivariate multi-step LSTM forecasting model for tuberculosis incidence with model explanation in Liaoning Province, China

Enbin Yang[1,2], Hao Zhang[1,2,3], Xinsheng Guo[1,2], Zinan Zang[1,2], Zhen Liu[1,4], Yuanning Liu[1,2,3*]

[1]College of Computer Science and Technology, Jilin University, 130012, Changchun, China.
[2]Key Laboratory of Symbolic Computation and Knowledge Engineering of Ministry of Education, Jilin University, 130012, Changchun, China.
[3]College of Software, Jilin University, 130012, Changchun, China.
[4]Graduate School of Engineering, Nagasaki Institute of Applied Science, 536 Aba-machi, 851-0193, Nagasaki, Japan.
*Correspondence: liuyn@jlu.edu.cn

List and Introduction of Additional Files

1. Supplementary Information: this word document includes an introduction to the full names of the factors, as well as additional figures of the model solution.
2. Complete data after processing: the data set used for the prediction model in this paper.
3. Raw meteorological data: 15 sets of raw meteorological data were obtained from seven monitoring stations.
4. Raw economic and social data: 9 sets of raw economic and social data.

When we solved the model, all the factors were given a variable name to simply express the meaning of the factors, as in **Supplementary Table T1**. Among them, each meteorological data was taken from seven meteorological monitoring stations in Liaoning province, which are Changwu, Chaoyang, Shenyang, Benxi, Yingkou, Dandong, and Dalian. The additional files "*raw_economic_and_social_data.xls*" and "*raw_meteorological_data.xls*" store the raw values of all the factor data. values for all factors, however, there are many missing values.

We gave complete results for all models, including the ARIMA model (**Supplementary Table T2**), SARIMA model (**Supplementary Table T3**), multivariate multi-step LSTM model (**Supplementary Table T4**), and multi-step ARIMA-LSTM hybrid model (**Supplementary Table T5**). These findings may be helpful in tuning the model parameters.

Supplementary Table T1 Variable names and factor names

Factor Category	Variable Name	Factor Name (unit)
Meteorological data	avr_air_pressure	Average air pressure (hPa)
	avr_wind_spd	Average 2-minute wind speed (m/s)
	avr_temp	Average temperature (°C)
	avr_water_pressure	Average water pressure (hPa)
	rel_humidity	Average relative humidity (%)
	day_precipitation	Number of days with daily precipitation ≥ 0.1mm (days)
	month_sun	Monthly sunshine percentage (%)
	hour_sun	Sunshine hours (h)
	extreme_wind_speed	Extreme wind speed (m/s)
	min_air_pressure	Minimum air pressure (hPa)
	min_temp	Minimum temperature (°C)
	max_air_pressure	Maximum air pressure (hPa)
	max_temp	Maximum temperature (°C)
	max_wind_speed	Maximum wind speed (m/s)
	day_max_precipitation	Maximum daily precipitation (mm)
Economic and social data	power	Electricity generation (billion kWh)
	industrial_add	Value added of industries above the scale (Year-on-year growth %)
	sales_rate	Industrial product sales rate (%)
	fin_budget	Local budget revenue (cumulative growth %)
	cpi_rural	Rural Consumer Price Index
	cpi_urban	Urban Consumer Price Index
	cpi	Average Consumer Price Index
	pas_turn_road	Road passenger turnover
	pas_turn_water	Waterway passenger turnover
	pas_turn	Average passenger turnover
	im_ex	Total value of imports and exports to and from domestic destinations/sources (thousands of USD)
	residential_investment	Residential investment volume (Cumulative year-on-year %)
	ex_factory_index	Ex-factory price index of industrial products

Supplementary Table T2 Prediction performance of the ARIMA models

$(\boldsymbol{p}, \boldsymbol{d}, \boldsymbol{q})$	**Ahead Size**	**RMSE**	**MAE**	**MAPE (%)**	**sMAPE (%)**
$(5,1,5)$	6	0.3736	0.2865	6.2383	5.9174
$(3,1,3)$		1.1175	0.9241	20.1837	17.7453
$(2,1,4)$		0.3244	0.2811	6.0454	5.8097
$(2,1,1)$		0.5164	0.4724	10.0354	10.4617
$(3,1,5)$		0.7241	0.5659	12.3576	11.2302
$(5,1,5)$	12	0.5300	0.4398	10.8737	10.0603
$(3,1,3)$		1.3026	1.1277	26.9630	22.9895
$(2,1,4)$		0.4425	0.3917	9.7674	6.0454
$(2,1,1)$		0.5250	0.4692	11.1693	11.7354
$(3,1,5)$		0.9007	0.7648	18.4558	16.3897
$(5,1,5)$	24	0.5628	0.4724	11.1506	10.3224
$(3,1,3)$		1.3423	1.1488	26.5904	22.5749
$(2,1,4)$		0.4672	0.4177	9.9328	9.3198
$(2,1,1)$		0.8994	0.7413	16.9383	19.3824
$(3,1,5)$		0.9290	0.7617	17.8239	15.7419

Supplementary Table T3 Prediction performance of the SARIMA models

$(\boldsymbol{p}, \boldsymbol{d}, \boldsymbol{q}) \times (\boldsymbol{P}, \boldsymbol{D}, \boldsymbol{Q})_{12}$	Ahead Size	RMSE	MAE	MAPE (%)	sMAPE (%)
$(0,1,1) \times (0,1,1)_{12}$	6	1.0935	1.0289	22.0253	19.5980
$(1,1,1) \times (0,1,1)_{12}$		1.0157	0.9339	20.0035	17.9006
$(0,1,2) \times (0,1,1)_{12}$		1.0287	0.9493	20.3302	18.1746
$(0,1,1) \times (1,1,1)_{12}$		1.0625	0.9988	21.3799	19.0798
$(1,1,2) \times (0,1,1)_{12}$		1.0963	1.0265	21.9627	19.5315
$(0,1,1) \times (0,1,1)_{12}$	12	0.8587	0.7368	16.5854	14.9808
$(1,1,1) \times (0,1,1)_{12}$		0.7825	0.6508	14.5400	13.2301
$(0,1,2) \times (0,1,1)_{12}$		0.7955	0.6651	14.8807	13.5211
$(0,1,1) \times (1,1,1)_{12}$		0.8354	0.7182	16.1828	14.6582
$(1,1,2) \times (0,1,1)_{12}$		0.8447	0.7109	15.8715	14.3506
$(0,1,1) \times (0,1,1)_{12}$	24	0.8471	0.7204	15.9791	14.5258
$(1,1,1) \times (0,1,1)_{12}$		0.7634	0.6384	14.1518	13.0495
$(0,1,2) \times (0,1,1)_{12}$		0.7778	0.6519	14.4502	13.2851
$(0,1,1) \times (1,1,1)_{12}$		0.8323	0.7083	15.7193	14.3164
$(1,1,2) \times (0,1,1)_{12}$		0.8040	0.6764	14.9867	13.7548

Supplementary Table T4 Prediction performance of multivariate multi-step LSTM models (17 factors)

Step Size (n)	Ahead Size	RMSE	MAE	MAPE (%)	sMAPE (%)
1	6	0.3296	0.2959	6.3009	6.2957
2		0.2825	0.2363	5.0797	4.9490
3		0.3713	0.3310	7.0924	6.7762
4		0.4272	0.3753	7.9774	7.5864
1	12	0.4614	0.3488	8.8951	8.3512
2		0.4060	0.3073	7.8076	7.5203
3		0.4838	0.3758	9.4591	8.7572
4		0.4617	0.3740	9.1130	8.7787
1	24	0.4499	0.3398	8.3577	7.9290
2		0.4108	0.3295	7.7436	7.4895
3		0.4632	0.3671	8.7567	8.2718
4		0.5297	0.4306	9.9386	9.7692

Supplementary Table T5 Prediction performance of multi-step ARIMA-LSTM hybrid models

Step Size (n)	Ahead Size	RMSE	MAE	MAPE (%)	sMAPE (%)
1	6	0.5716	0.4063	8.6308	9.4558
2		0.5850	0.4444	9.3583	10.1987
3		0.4659	0.3206	6.8661	7.4156
4		0.4731	0.3627	7.7163	8.2338
1	12	0.4423	0.2996	6.9118	7.3411
2		0.4438	0.3081	6.9633	7.4099
3		0.3753	0.2619	6.1742	6.4240
4		0.3913	0.2868	6.7508	6.9308
1	24	0.4508	0.3352	7.5220	7.8815
2		0.4787	0.3489	7.7385	8.1999
3		0.4042	0.3070	6.9664	7.2245
4		0.4208	0.3254	7.3678	7.6021

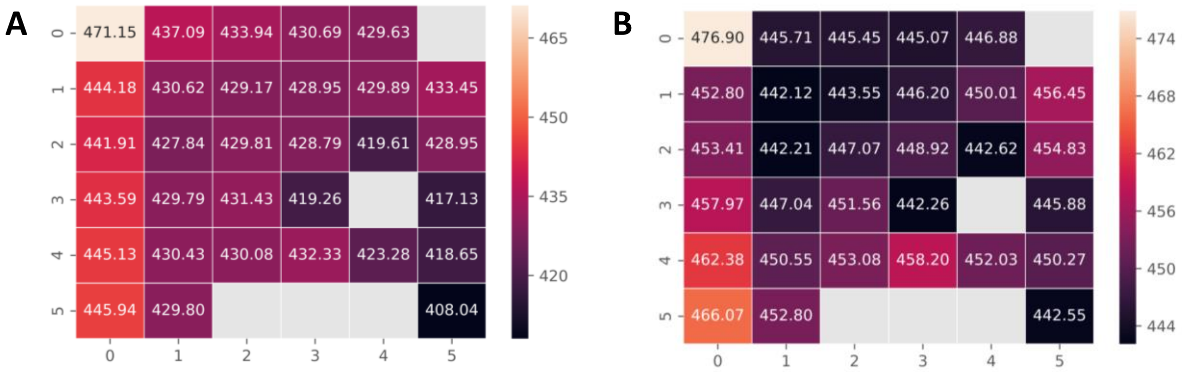

Supplementary Figure S1 AIC and BIC heat maps of the ARIMA model

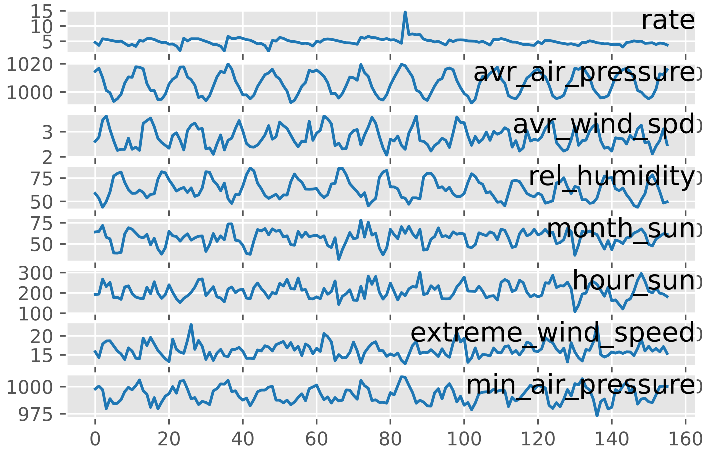

Supplementary Figure S2 Relationship between morbidity and various factors
